# Effects of self-compassion on aggression and its psychological mechanism through perceived stress

**DOI:** 10.1186/s40359-024-02191-w

**Published:** 2024-11-16

**Authors:** Fang Guan, Chengqing Zhan, Shanyin Li, Song Tong, Kaiping Peng

**Affiliations:** 1 School of Psychology, Army Medical University, Chongqing, China; 2 Department of Psychological and Cognitive Sciences, Tsinghua University, Beijing, China

**Keywords:** Self-compassion, Reactive aggression, Perceived stress, Mental health interventions, Chinese adults

## Abstract

Given the increasing global concerns about aggressive behaviors and the pressing need for effective psychological interventions, this study delves into the potential of a concept deeply rooted in positive and Buddhist psychology but largely researched in Western contexts, as a mitigating factor against aggression in Chinese adults. Through three core studies involving 652 participants (age: 30.52±8.16), our research illuminated the intricate relationship between self-compassion, perceived stress, and aggression. Study 1 identified a negative correlation among these variables, setting the empirical foundation. In Study 2, participants exposed to a self-compassion exercise reported enhanced self-compassion and reduced aggression. Study 3 further consolidated these findings, with participants in the self-compassion writing group, showing notable increases in self-compassion and decreases in aggression compared to a control group. Critically, perceived stress emerged as a significant mediator between self-compassion and aggression, elucidating its central role in this dynamic. Together, our findings underscore the promise of self-compassion as a strategy to curb aggression tendencies, especially in light of its influential relationship with perceived stress, suggesting vital implications for future mental health interventions.

## Introduction

Self-compassion, anchored in the principles of self-care and empathy, has recently emerged as a focal point due to its capacity to shape individuals’ emotional reactions and behavioral patterns [1, 2]. While much of the discourse on self-compassion has centered on internalizing symptoms, its ties to externalizing symptoms remain comparatively underexplored. The current research endeavors to elucidate the transition from self-compassion to actions of compassion, with an emphasis on reducing externalizing aggression. By investigating the psychological underpinnings bridging this relationship, we aspire to deepen our grasp of emotional regulation in humans and unveil new pathways for specialized mental health interventions.

Building upon the theoretical framework by Neff (2003) [1], self-compassion emerges as a positive response individuals embraces during times of distress. This concept is tri-faceted, comprising: (a) self-kindness, characterized by extending understanding and warmth towards oneself during hardship; (b) common humanity, which emphasizes the universality of human struggles, fostering a feeling of interconnectedness; and (c) mindfulness, representing balanced and non-judgmental recognition of distressing emotions and thoughts. The Self-Compassion Scale (SCS) distinctly measures these dimensions against their opposing elements: self-kindness versus self-judgment, common humanity versus isolation, and mindfulness versus over-identification [1, 3]. This intricate construct sheds light on individual coping strategies during adversity, especially concerning emotional regulation, and monitoring and the evaluation of ongoing behaviors [4, 5]. Mounting evidence suggests that higher self-compassion is inversely related to emotions like anger, anxiety and depression [6–10]. Furthermore, it’s been linked to reduced negative affect [11], diminished perceived stress [4, 8], and heightened overall life satisfaction or well-being [12–14].

While a significant portion of the self-compassion literature has focused on its influence on internalizing symptoms, there’s an emerging interest from Western researchers exploring its potential ramifications on aggression. Aggression is generally defined as a behavior whose purpose is to harm an individual who is perceived to want avoid being harmed [15], which can take different forms [16, 17]. Human aggression is highly prevalent and has a great impact on the lives of victims and on society as a whole [18]. Although different researchers defined aggression as different behaviors, like antisocial behavior [19] or disrupting behavior [20], accruing evidence suggested that aggression was associated with violence and criminal activities [21], mental health issues [18], and malevolent creativity [22]. Recently, more attention has been paid to reveal the relationship between stressful life events and different forms of aggression. It is believed that perceived stress or the assessment of stressful life events is closely related to aggression, especially for reactive aggression [23].

Empirical evidence indicates that self-compassion maybe a protective factor predicting distinct manifestations of aggression. For example, among adolescent males at risk, a notable association was observed between heightened self-compassion and a decrease in both reactive and proactive forms of aggression [16]. Furthermore, empirical findings suggest that individuals of both genders, when exhibiting diminished self-compassion, tend to manifest higher tendencies towards verbal aggression with their romantic relationships [24]. Recent studies have highlighted the efficacy of interventions aiming to augment self-compassion, suggesting their potential role in mitigating aggressive externalizing behaviors [25, 26].

Grounding this exploration in Berkowitz’s Associative Network Model of Aggression [27, 28], the manifestation of externalized aggressive syndromes frequently interlinks with the perception of stress [25, 29–31]. A revealing longitudinal study postulated that adolescents experiencing elevated levels of academic, parental, and peer stress were more predisposed to engage cyber aggression, particularly when intersecting with instances of cyber victimization [32]. Additionally, previous study also provided evidence from the aspects of asymmetric frontal brain activity, suggesting this lateralized brain activity elicited by stress exposure could predict an increase in subsequent aggressive behavior [33]. Significantly, the role of perceived stress in determining emotion regulation strategies, like self-compassion, cannot be understated [5]. A notable meta-analysis by Ferrari et al. (2019) [34] emphasized a substantial link between self-compassion and stress, with a confidence interval suggesting a moderate yet consistent relationship (g = 0.67; 95% CI = 0.37–0.96). Furthermore, a comprehensive cross-sectional research involving 1,453 Chinese nursing students indicated that self-compassion indirectly affects anxiety and depression through perceived stress [8]. Highlighting the broad impact of self-compassion on medical adherence, a revealing meta-analysis deduced that approximately 11% of the variance in adherence, attributable to self-compassion, could be linked to a decrease in perceived stress [4]. Thus, the critical role of perceived stress emerges as a potential conduit, bridging self-compassion with aggression reduction.

Nevertheless, while a substantial amount of research on the influence of self-compassion on individual behavior stems from Western industrialized contexts [35, 36], its roots trace back to Buddhist psychology [37]. Interestingly, there’s a paucity of studies examining the ramifications of self-compassion within East Asian countries. Particularly in nations like China, there’s a notable gap in empirical studies exploring the impact and efficacy of self-compassion-based interventions on behavior. Furthermore, empirical research has indicated a concerning increase in the prevalence of aggression, posing a significant threat to public health [38], especially in the aftermath of the prolonged COVID-19 pandemic [39]. Given the current global scenario and cultural nuances, there is an urgent call to evidence-based psychological interventions suitable for broad implementation in the post-epidemic era, with a focus on Eastern contexts.

Consequently, the primary objectives of this research are (1) to delineate the relationship between self-compassion and aggression in the context of Eastern culture, which provide a precondition for demonstrating how perceived stress may mediate the relationship between self-compassion and aggression, while assessing the potential mediating effects of perceived stress within the context of Eastern culture, (2) to assess the efficacy of self-compassion-based intervention in curbing aggressive intentions or behaviors. This research is systematically divided into three pivotal studies to probe the nexus between self-compassion and aggression. Study 1 sets the empirical groundwork by analyzing the interplay between self-compassion, perceived stress, and aggression. Study 2, employing a randomized experimental design, evaluates the impact of self-compassion writing exercises on aggression diminution, offering tangible evidence for the potential benefits of self-compassion-based interventions. In Study 3, using a randomized controlled design, we delve deeper into the effects of self-compassion writing on aggression reduction while simultaneously examining the mediating role of perceived stress. This layered exploration aims to provide an in-depth insight into the dynamics between self-compassion and aggression.

## Study 1

The primary objective of Study 1 was twofold. Initially, we sought to validate the already recognized negative correlation between self-compassion and perceived stress, alongside the positive association between perceived stress and aggression trait, as corroborated by earlier research [4, 8, 32]. Simultaneously, we were keen to discern any negative correlations between self-compassion and aggression, while evaluating the potential mediation effect of perceived stress within a Chinese general public sample.

### Method

#### Participants

After eliminating 10 participants who failed the lie detector test or presented regular answers, the sample consisted of 315 participants recruited from Credemo platform^1^ ($M_{age}$ = 31.06, SD = 7.72, Range = 18–56). Among the participants, approximately 71.4 % (n=225) of the sample identified as female, 28.6 % (n=90) as male. The majority of participants (96.4%) self-reported as belonging to the Han ethnic group. They completed the study for 10 Yuan per person.

#### Procedure

Participants were recruited from the online Credemo platform, an internationally recognized questionnaire collection platform, and paid for their participation. Prior to the study, informed consent was obtained from all participants. All procedures performed were in accordance with the APA Ethical Principles and the standards established by the authors’ Institutional Review Board.

#### Materials

**Self-Compassion Scale (SCS)**: The self-compassion scale [1] consists of 26 items assessing how individuals relate to themselves during times of sufferings. As defined by Neff (2003) [1], self-compassion contains three positive components and their corresponding negative counterparts, which together form a self-compassionate response (including emotional, cognitive, and attentive aspects). Participants rated each item on a 5-point Likert scale, ranging from 1(almost never) to 5(almost always). Examples of worded items reflecting self-compassion are “I try to be loving toward myself when I’m feeling emotional pain”. To yield an overall self-compassion score, the negative items representing were reverse-coded, and then mean subscale scores were averaged. The Chinese version of the SCS has been found to demonstrate good reliability and validity among Chinese adults [11, 40]. The internal consistency was also excellent in the current study (Cronbach’s $\alpha$ = 0.85).

**Perceived Stress Scale (PSS)**: Participants completed a self-report 14-item version of the perceived stress scale [41], which is a well-established measure of general stress levels. The scale assesses perceived stressful feelings and thoughts experienced within the past month and employs a five-point Likert-type scale, with response options ranging from “0=never” to “4=very often”. PSS scores are obtained by reversing the scores on the seven positive items and then summing across all 14 items. Higher scores indicate high levels of perceived stress. Previous evidence has demonstrated satisfactory psychometric properties of the PSS in Chinese populations [42], and in the current study, the PSS scores exhibited good internal consistency (Cronbach’s $\alpha$ = 0.77).

**Buss-Perry Aggression Questionnaire (BPAQ)**: The aggression questionnaire [43] consists of 29 items designed to assess four aspects of human aggression, including physical and verbal aggression, anger, and hostility. In the current study, we employed the Chinese College Buss-Perry Aggression Questionnaire developed by Li et al. (2011) [44], which was based on Buss and Perry’s (1992) [43] aggression questionnaire. The Chinese version also contains four subscales, namely, physical aggression (5 items), impulsivity aggression (6 items), anger proneness (3 items), and hostility (8 items). Participants were asked to rate each item using a 5-point Likert format (1= extremely uncharacteristic of me, 5 = extremely characteristic of me). In the current study, the Chinese College Buss-Perry Aggression Questionnaire demonstrated excellent internal consistency (Cronbach’s $\alpha$ = 0.89).

### Results and discussion

**Common method bias**: EFA (Eloratory factor analysis) was employed to access common method bias [45]. The interpretation of the probability of the first common factor was 21.56%, which is far less than the 40% threshold. Therefore, there was no substantial common method bias in the study 1.

**Descriptive statistics and correlations among variables**: Table 1 presented the descriptive statistics and correlations for the variables in the current study. The self-compassionate scores exhibited negative associations with perceived stress, and aggression. In addition, Pearson correlation analyses showed that self-compassion was negatively associated with all four aspects of human aggression, while perceived stress was positively associated with all four aspects of human aggression.

**Table 1:** Descriptive statistics and correlations among study variables

Variables	1	2	3
1. Self-compassion	1		
2. Perceived stress	−0.69**	1	
3. Aggression	−0.65**	0.62**	1
Mean	4.21	0.90	1.98
SD	0.37	0.38	0.54

** $$p < 0.01$$

**Fig. 1:**
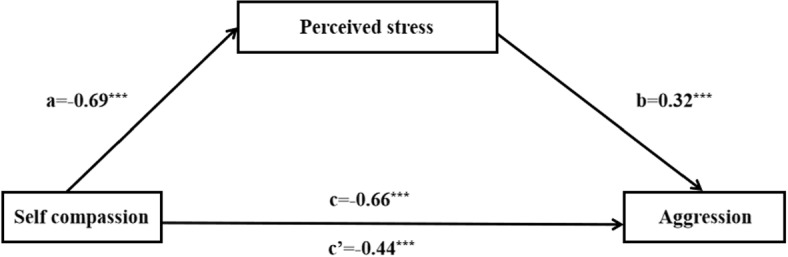
Mediation models showing the effect of self-compassion as mediated by perceived stress, on aggression in Study 1. Standard regression coefficients are shown. Asterisks indicate significant results (*** $p < .001$)

**Mediation via perceived stress**: The mediation analysis was conducted using PROCESS ([46]; Model 4), with bootstrap confidence intervals not including zero indicating a significant effect. As expected by our hypothesized model (see Fig. 1), self-compassion negatively predicted participants’ perceived stress (${\upbeta }$ =−0.69, 95% CI $[-0.77, -0.61]$). Subsequently, perceived stress positively predicted aggression (${\upbeta }$ = 0.32, 95% CI [0.21, 0.43]). Importantly, the results revealed a significant indirect effect of self-compassion on aggression through perceived stress (${\upbeta }$ = −0.22, SE = 0.04, 95% CI [−0.31, −0.14]), after controlling for age and sex.

It was noting that, Pearson correlation analyses showed that four aspects of human aggression was associated with both self-compassion (negatively) and perceived stress (positively). However, physical and verbal attacks represent direct forms of aggression, while hostility and anger signify cognitive and emotional dimensions of aggression. Participants in Study 1, averaging around 30, were less inclined to exhibit overt direct aggression, a trend typical of at the adult stage. Instead, relational and indirect forms of aggression are more common. The pattern may be attributed to empirical findings suggesting a notable rise in the prevalence of aggression, especially in the aftermath of the prolonged COVID-19 pandemic [39].

In spite of this, the findings from Study 1 provided support the interrelationship of the study variables and proposed mediation model, indicating that perceived stress might mediate the relationship between self-compassion and aggression. However, it is important to note that the correlational design of the Study 1 limits the ability to draw causal inferences. Therefore, subsequent experimental studies were designed to explore whether self-compassion-based intervention can effectively reduce individuals’ aggression, particularly through the mechanism of reduced perceived stress.

## Study 2

Having established a notable negative link between individual self-compassion and aggression in Study 1, the objective of Study 2 was to assess initial effectiveness of Self-Compassionate Mindstate Induction (SCMI), as adopted in Neff et al. (2020)’s research [47], to attenuating the participants’ aggressive tendencies. We hypothesized that those exposed to self-compassionate intervention would report diminished aggression scores in comparison to compared to their counterparts in the control group.

### Method

#### Participants

One hundred and seventy participants took part in the study on the Credemo platform. After excluding 8 participants who failed the compliance check, 162 participants (Mage=29.9, SD=9.35, 62 males, 100 females) were retained for subsequent data analyses. The self-compassion intervention group consisted of 81 participants (Mage=30.80, SD=9.12), while the control group was also composed of 81 participants (Mage=28.93, SD=9.56). As an expression of gratitude, participants who completed the study were thanked and received monetary compensation (RMB 10 Yuan) for their participation.

#### Procedure

All participants were recruited from the Credemo platform, and after obtaining informed consent, they were asked to provide basic demographic information. Subsequently, they were randomly assigned to one of two conditions: (1) self-compassion writing induction condition and (2) neutral control condition. In the next step, participants completed a brief survey where they were instructed to think about a particular situation, they found difficult. Participants were then asked to rate, on a 7-point Likert scale (ranging from 1=a little difficult to 7=extremely difficult), how difficult the perceived the situation to be. The majority of participants selected a fairly difficult situation (M=5.7, SD=1.01) to contemplate. Then, all participants from both groups were instructed to complete a writing task about the same situation based on the survey’s instructions. Following the completion of the writing tasks, participants answered a compliance check question, and the proceeded to fill out the relevant measures.

#### Materials

**Self compassion writing condition**: Participants assigned to the self-compassion intervention group engaged in a writing task that consisted of a series of prompts designed to induce the three components of self-compassion: mindfulness, common humanity, and self-kindness. The Self-Compassion Writing procedure closely followed the Neff et al. (2020)’s research [47], with the only modification being the replacement of the “Thanksgiving” with a Chinese national holiday “The Dragon Boat Festival”. The whole writing task was already used in our prior research (for more details, referred to [11]).

**Neutral control condition**: The neutral control condition was carefully designed to be parallel to the Self-Compassion Writing condition. Following Neff’s procedures, participants assigned to the control condition were instructed to begin by writing about the painful situation in a purely descriptive manner (parallel to mindfulness). Subsequently, they were asked to identify and specify the individuals involved int the situation (parallel to common humanity) and describe any words spoken in the situation (parallel to self-kindness). This approach ensured that both the self-compassion intervention group and the control group engaged in similar writing tasks, while the content and focus of the writing prompts differed to test the effectiveness of the self-compassion intervention.

**Compliance check**: At last, all participants were asked to indicate the task they had just completed during the writing task. They were presented with two potions and required to select one. In the self-compassion writing condition, participants passed the compliance check if they responded with“a” while in the neural control condition passed if they responded with“b”. Data of 8 participants who failed the compliance check were subsequently excluded from the analysis.

- (a) “write about your feelings in an accepting and validating way, consider how going through difficult situations is part of being human, write to yourself like a supportive friend”.
- (b) “write the details of the situation, who is involved and what was said with as much detail as possible”.

**State self compassion scale long form**: State Self-compassion Scale-Long Form (SSCS-L) [47], containing 18 items, was employed to assess individuals’ self-compassion levels during the data collection period. An example item includes “I’m remembering that there are lots of others in the world feeling like I am”. Participants were asked to rate each item on a 5-point Likert scale, ranging from 1= “not at all true for me” to 5= “very true for me”. Before generating an overall self-compassion score, items representing self-judgment, isolation, and over-identification were reverse-coded. Higher scores on the SSCS-L represented higher levels of state self-compassion. The scale has demonstrated robust psychometric properties both in the West culture [47] and Chinese culture [11]. In this study, the scale showed good internal reliability ($\alpha$=0.92).

**State aggression**: The Shooting Game Questionnaire (SGQ), adapted from Russell et al. (1996) [48] and utilized in previous Chinese research by Yang et al. (2016) [40], was employed in this study to assess aggressive behavior. The instructions were as follows:

“*After the experiment, you can participate in a shooting game in the zoo where you can shoot at a bear. The bear is in an iron cage and it cannot escape. You can choose one of seven toy guns that have been reduced in power, but still vary in strength. For example, Gun #1 is fairly weak, Gun #4 is about average, and Gun #7 is fairly strong. You can take between one and seven shots at the bear. Now, please choose the power of the gun and the number of bullets you will shoot.*”

### Data analyses

Data analyses were performed using SPSS Statistics Version 25.0, and the necessary statistical assumptions were checked. Preliminary analyses indicated that variables did not exhibit significant differences based on gender in both the self-compassion and control groups. However, age showed a positive correlation with self-compassion scores and aggression-gun scores. Consequently, only participants’ age was included as a covariate in subsequent analyses to account for its potential influence on the results.

### Results and discussion

The results of the independent sample t-tests are presented in Table 2. It is evident that participants in the self-compassion writing condition reported significantly higher levels of self-compassion perception compared to participants in the control condition. Consequently, participants in the self-compassion condition exhibited lower level of aggression-gun score in comparison to the participants in the control condition. However, no significant difference was found in aggression-shots score between the two groups. The possible explanation maybe that, while the SGQ is a validated measure of aggressive behavior, it posed limitations as an aggression assessment tool due to some participants perceived the scenarios overly violent, as indicated by [40]. More choices of bullets lead to longer shooting duration and increased brutality. Consequently, alternative methods will be employed to measure aggression and enhance the experimental design in our future study. Nevertheless, these findings offer partial support to past studies suggesting self-compassion writing can effectively diminish individuals’ aggression. Nevertheless, the findings offer partial support to past studies suggesting self-compassion writing can effectively reduce individuals’ aggression.

**Table 2:** Means and SDs of main study variables by group condition in study 2

	Compassion		Control		*t*	Cohen’s *d*
	Mean	SD	Mean	SD		
Self-compassion	72.37	9.23	64.19	13.51	4.50$$^{***}$$	0.71
Aggression (gun)	2.64	2.15	3.52	2.49	−2.40$^{*}$	0.38
Aggression (shots)	2.42	1.86	2.73	2.10	0.32	0.16

* $p<.05$, *** $p<.001$. Bootstrapping with 5000 resamples were applied

## Study 3

The results of Study 2 demonstrated a promising effect of the self-compassion intervention in alleviating individual’s aggression. However, the main limitation of the study was the absence of pre-test measures, which prevented an accurate estimation of the self-compassion intervention’s true effect. Additionally, while the SGQ is a validated measure of aggressive behavior [40], it posed limitations as an aggression assessment tool due to some participants finding the scenario too violent. In addition, we also sought to examine whether perceived stress would mediate the expected relationship between self-compassion induction and aggression, and to conceptually replicate, the way of experiment, cross-sectional results in Study 1 evidencing that a perceived stress mediated the relationship between self-compassion and aggression.

### Method

#### Participants

The participants in this study were recruited from a diverse range of backgrounds to ensure a representative sample in Credemo. Referred to our previous research, a priori power analysis using the G*Power 3.1.9.2 [49] determined that a total sample of no fewer than 54 participants was required [11]. 180 participants between the ages of 19 and 56 (M = 30.29, SD =7.44) participated in the research. Five participants were excluded because of failing to pass the compliance check. Data on the remaining 175 participants (102 females, 73 males) were included in the next analysis. Participants provided informed consent before their involvement and were compensated RMB 15 Yuan for their time. Confidentiality and anonymity of participants’ responses were strictly maintained throughout the study.

#### Procedure

The study was conducted using the Credemo platform. Prior to accessing the survey, participants were presented with a consent form outlining the study’s objectives, procedures, and data confidentiality. By proceeding, participants indicated their informed consent. Upon accessing the survey, participants were first asked to provide demographic information, such as age, gender, and educational background. Subsequently, they were presented with a series of self-report pre-test measures (i.e., SSCS-L, PSS) regarding the situation. Then, participants were randomly allocated to one of two experimental conditions like Study 2: (1) self-compassion writing induction condition and (2) neutral control condition. After completing the writing tasks, participants answered the compliance check question and filled out the post-test measures. Once the survey was completed, participants were thanked for their participation and provided with compensation in accordance with Credemo guidelines.

#### Materials

**Self compassion/Neutral control induction**: The same with Study 2.

**State self compassion scale long form**: The same with Study 2. In this study, Cronbach’s $\alpha$ was 0.93 at pre-test and was 0.94 at post-test.

**Compliance check**: The same with Study 2. Data of 5 participants who failed the compliance check were subsequently excluded from the analysis.

**Perceived stress scale**: Participants completed a self-report 14-item version of the perceived stress scale [41], akin to that employed in Study 1. Unlike the Study 1, the scale in Study 3 assessed perceived stressful feelings and thoughts experienced in here and now and employed a five-point Likert-type scale, with response options ranging from “1=not at all” to “5=very obvious”. PSS scores are obtained by reversing the scores on the seven positive items and then summing across all 14 items. Higher scores indicate high levels of perceived stress. The PSS was found to be reliable at pre-test ($\alpha$=0.91) and at post-test ($\alpha$=0.92).

**The Reactive-Proactive Aggression Questionnaire (RPQ)**: The RPQ is a well-established self-report measure designed to assess different forms of aggression in individuals [50]. It consists of separate subscales for reactive aggression, characterized by impulsive and hostile reactions to perceived provocation, and proactive aggression, characterized by planned and instrumental aggression aimed at achieving desired goals. Participants are asked to rate the extent to which each item describes their thoughts, feelings, and behaviors on a Likert-type scale, ranging from 1 (strongly disagree) to 7 (strongly agree). Higher scores on each subscale indicate higher levels of reactive or proactive aggression. The RPQ has demonstrated good reliability and validity across various populations and cultural contexts (e.g., [45, 51]. In this study, Cronbach’s $\alpha$ was 0.93 at pre-test and was 0.93 at post-test.

### Results and discussion

Table 3 displayed the means and standard deviations of the main study measures at both pre-test and post-test. At the pre-test, no statistically significant differences were found between the self-compassion writing group and control group on all study variables (all *p* >0.05). The paired t-test results for pre/post-test outcomes indicated significant increases in the self-compassion writing group’s level of self-compassion (*t*=−5.13, *p* <0.001), and significant decreases in perceived stress (*t*=3.57, *p* <0.001) and aggression (*t*=6.04, *p*=0.001). In comparison, none of the pre/post-test changes in the control condition reached statistical significance (all *p* >0.05)in the neutral control group.

**Table 3:** Pre-test and post-test mean scores by condition and effects of the self-compassionate writing intervention using 2 (Condition) X 2 (Time) repeated measures analyses of variance in study 3

	Self-compassionate writing		Neutral control		*F*	$\eta ^2_p$
Outcome	Pretest	Posttest	Pretest	Posttest		
Self-Compassion	72.90 (10.75)	77.02 (7.67)	73.43 (12.73)	69.99 (16.16)	16.06***	.09
Perceived Stress	32.49 (10.91)	29.66 (9.34)	32.19 (11.84)	34.50 (13.16)	11.96**	.02
Aggression	52.98 (19.62)	43.88 (15.96)	53.33 (21.04)	51.12 (21.86)	6.29*	.04

*$$p < .05$$, ** $$p < .01$$, *** $$p < .001$$

To assess the extent of reduction in participants’ aggression within the self-compassion writing group, we conducted a 2$\times$2 repeated-measures ANOVA with Condition (self-compassion writing and control writing) as a between-subjects factor and Time (pre-test and post-test) as a within-subjects factor. As shown in Table 3, participants in the self-compassion writing condition exhibited significantly greater increases in self-compassion and greater reductions in their level of perceived stress and different forms of aggression compared to those in the control condition.

**Fig. 2:**
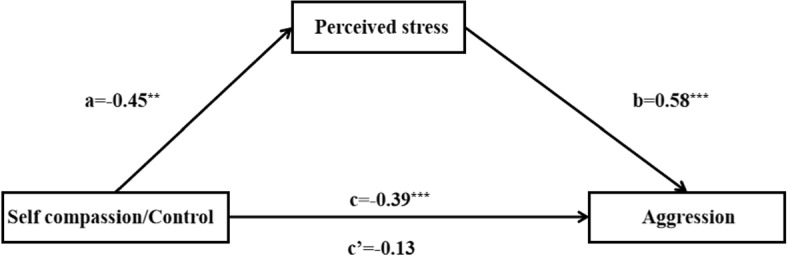
Mediation models showing the effect of condition (Self-compassion vs. Control), as mediated by perceived stress, on reactive aggression in Study 3. Standard regression coefficients are shown. Condition is dummy-coded (Self-compassion=1, Control=0). Asterisks indicate significant results (*$p < .05$, **$p < .01$,***$p < .001$)

As reported above, the self-compassion intervention led to decreased perceived stress and different forms of aggression. In addition, the perceived stress was positively correlated with aggression (*r* =.32, *p* <.01) and reactive aggression (*r* =.42, *p* <.01) in the intervention task. Therefore, mediation analyses were conducted to assess the potential effect of the self-compassion induction on reducing aggression and reactive aggression, through perceived stress. Furthermore, preliminary analyses indicated that variables did not exhibit significant differences based on gender in both the self-compassion and control groups. However, age showed a negative correlation with aggression and perceived stress scores. Consequently, only participants’ age was included as a covariate in subsequent analyses to account for its potential influence on the results. Figure 2 illustrated the mediation model and presents the path coefficients. The outcomes demonstrated that only the relationship between self-compassion and reactive aggression could be mediated by perceived stress. Utilizing the bootstrapping procedure for mediator models [52, 53] with 10,000 bootstrap iterations, indirect effect coefficients (−0.27), BootSE (0.09), and a 95% bias-corrected confidence interval (−0.45 to −0.10) was obtained, excluding zero, after controlling for age. This analysis indicated that self-compassion contributes to the mitigation of reactive aggression by perceived stress.

Indeed, the present findings align with prior research suggesting self-compassion writing interventions have the potential to reduce individuals’ aggression [34]. Furthermore, with the enhancements made in the intervention design for study 3, the results presented more robust evidence supporting the effectiveness of self-compassion-based practices in reducing reactive aggression through perceived stress, which was consistent with previous studies [23, 54]. The findings from this study contribute to the growing body of literature on the beneficial effects of self-compassion interventions in promoting healthier emotional regulation and reducing aggressive behaviors. It underscores the value of self-compassion-based approaches as valuable tools in addressing and mitigating aggression in various contexts.

## General discussion

The current investigation aimed to establish connections between self-compassion and aggression among Chinese individuals, with an emphasis on determining whether self-compassion-based intervention could reduce aggression by alleviating perceived stress. As anticipated, we found a significant negative association between individuals’ self-compassion and personality trait of aggression in Study 1, which connection could be mediated by perceived stress. Intriguingly, our subsequent experimental studies (Study 2 and 3) demonstrated that self-compassion writing exercise might hold promise in reducing aggression among the general population. Moreover, Study 3 further substantiated the mediating role of perceived stress in bridging self-compassion and reactive aggression.

In line with our initial hypothesis, we replicated the well-established negative correlation between self-compassion and perceived stress, and the positive correlation between perceived stress and aggression, as supported by previous studies [4, 8, 32]. More importantly, individuals with high self-compassion tended to report less personality trait aggression. This significant negative association with moderate-to-strong effect size was consistent with prior research studies, which found relations between self-compassion and various forms of aggression in Western culture [16, 24]. In addition, the finding of Study 1 supported the proposed mediation model, indicating that perceived stress might mediate the relationship between self-compassion and personality trait aggression. The findings suggested that both in Western and Eastern culture, self-compassion as a healthy self-attitude not only improve individuals’ internalizing psychological resources reported by previous research [55, 56], but also potentially dampens the manifestation of externalizing aggression.

In the aftermath of the prolonged COVID-19 pandemic, substantial evidence has indicated a marked increase in both stress and aggression among individuals [39, 45]. Given this context, the online dissemination of self-compassion writing exercises holds significant promise in harnessing personal resources and in reaching a wide spectrum of individuals. Our findings (Study 2 and Study 3) showed that online self-compassion writing exercise could successfully induce individuals’ self-compassion, which consistent with our previous research during the era of social distancing caused by COVID-19 [11]. Moreover, similar to past research that based self-compassion interventions, our results revealed that inducing self-compassion through writing exercise can reduce aggression (Study 2 and Study 3) and perceived stress (Study 3) [4, 25, 26]. Neff (2016) [3] has defined self-compassion as a multidimensional construct that can be broadly categorized into three key domains: individuals’ emotional responses to suffering (characterized by kindness or self-judgment), their cognitive understanding of their challenges (either as a shared human experience or as isolating), and their approach to attending to suffering (mindful or overly identified). These components of self-compassion have the potential to manifest in a gentle, nurturing manner, particularly when directed toward self-acceptance or the alleviation of distressing emotions [37]. Such positive attitudes, when transferred from oneself to others, could influence externalizing behaviors such as prosociality or aggression [34, 57, 58]. These findings suggested that self-compassion may be a healthy way of relating to one’s self motivated by a desire to help rather than harm.

Furthermore, the statistical mediation analysis indicated that perceived stress served as a mediator in the distinct relationship between self-compassion and aggression, encompassing both the personality trait of aggression and reactive aggression. Both the cross-sectional and experimental findings of the mediating model in the current study (Study 1 and 3) provides us with evidence that improving the level of self-compassion could help reduce perceived stress, thereby reducing aggression among general people. These outcomes align with component (mindfulness) of self-compassion’s observed negative link with stress, subsequently leading to decreased aggression [59]. Previous evidence showed that mindfulness (the third component of self-compassion) and self-compassion are interconnected constructs, as they both encourage embracing discomfort with an accepting mindset [60]. Research has demonstrated that self-compassion and mindfulness assessments exhibit a shared variance ranging from 13 to 48% [1, 25, 61]. These findings highlight the crucial role of both mindfulness and self-compassion in fostering more constructive responses to emotional challenges and promoting a reduction in aggressive tendencies, which was consistent with our findings that self-compassion induction could mitigate aggression in the current investigation.

Additionally, in exploring the relationship between self-compassion and different types of aggression (reactive and proactive aggression), results demonstrated that self-compassion could reduce both forms of aggression, which is consistent with previous research [16]. But in exploring the role of perceived stress in mediating self-compassion and two forms of aggression, perceived stress was merely found to mediate the relationship between self-compassion and reactive aggression. It’s not hard to understand, because as an emotion regulation strategy, self-compassion refers to how we relate to ourselves in instances of perceived failure, inadequacy, or personal suffering, requiring individuals with awareness, non-judging of inner experience, and non-reactivity to inner experience [37]. This inward peaceful power is able to influence outward behaviors [4, 57, 58], which is consistent with what we found in current studies that self-compassion induction could reduce both reactive and proactive aggression in individuals. However, we found no correlation between perceived stress and proactive aggression, resulting in the mediating model not being valid. Prior evidence suggested that although proactive and reactive aggression have overlapping features and often co-occur [62], they differ in their underlying emotional and motivational drivers and underlying mechanisms [63, 64]. Proactive aggression (often called “cold-blooded”) is described as offensive, and manipulative, goal-oriented, aiming to achieve benefits even in the absence of provocation or anger, whereas reactive aggression (often called “hot-blooded”) is defensive, affective, and typically triggered by perceived threats or provocations [65, 66]. Our findings are consistent with existing empirical research, which showed that the post-traumatic stress cluster related to intrusive symptoms has a substantial indirect influence on the association between child maltreatment and reactive aggression but not proactive aggression [67]. However, further work is still needed to understand the potential of self-compassion-based intervention for mitigating different forms of aggression among the general public.

### Limitations and future research

While our findings offer a fresh perspective, it’s important to consider their implications within the framework of certain limitations. Firstly, considering that our study relies on self-report survey data obtained from the same respondents, it’s important to acknowledge the potential presence of common method variance. This could arise from participants’ tendencies to provide socially desirable responses or be influenced by implicit theories [68]. Secondly, our findings primarily stem from the outcomes of single brief intervention sessions. The long-term efficacy of these self-compassion exercises on individuals’ externalizing behaviors remains uncertain. Future research could adopt a longitudinal approach to investigate self-compassion-based interventions, estimating and confirming their effects on aggression while exploring their potential benefits for various facets of externalizing intentions or behaviors. Thirdly, although the self-compassion-based intervention was able to reduce both reactive and proactive aggression, when testing whether perceived stress mediated the relationship between intervention condition and different forms of aggression, we found that the mediation model only held when aggression type was reactive aggression. Despite the plausible explanations we have given, future research needs to further explore the relationship between these variables and the stability of this association.

Overall, our findings demonstrated that self-compassion is associated with less aggression among general public sample, due in part to lower perceived stress. This research contributes to the growing body of literature on the potential benefits of self-compassion in fostering positive behavioral outcomes and addressing aggression [34, 58]. Further work is needed to gain a more nuanced perspective on how self-compassion-based intervention may be beneficial for different forms of aggression across different populations, and to test other potential explanatory pathways beyond perceived stress.

## Data Availability

The datasets used and analysed during the current study are available from the corresponding author on reasonable request.

## Footnotes

1 https://www.credamo.com/
